# Supplementary material for: The phased pan-genome of tetraploid European potato
Source: Nature. 2025 Apr 16;642(8067):389–97. doi: 10.1038/s41586-025-08843-0 (PMC12158759; doi:10.1038/s41586-025-08843-0)
Supplement: Supplementary file 5 — Supplementary Figs. 3–14, 20, 21, 23–27 and 34–44. [file 41586_2025_8843_MOESM5_ESM.zip › suppl_figure_34-44/suppl_figure_42_I_v3.pdf]

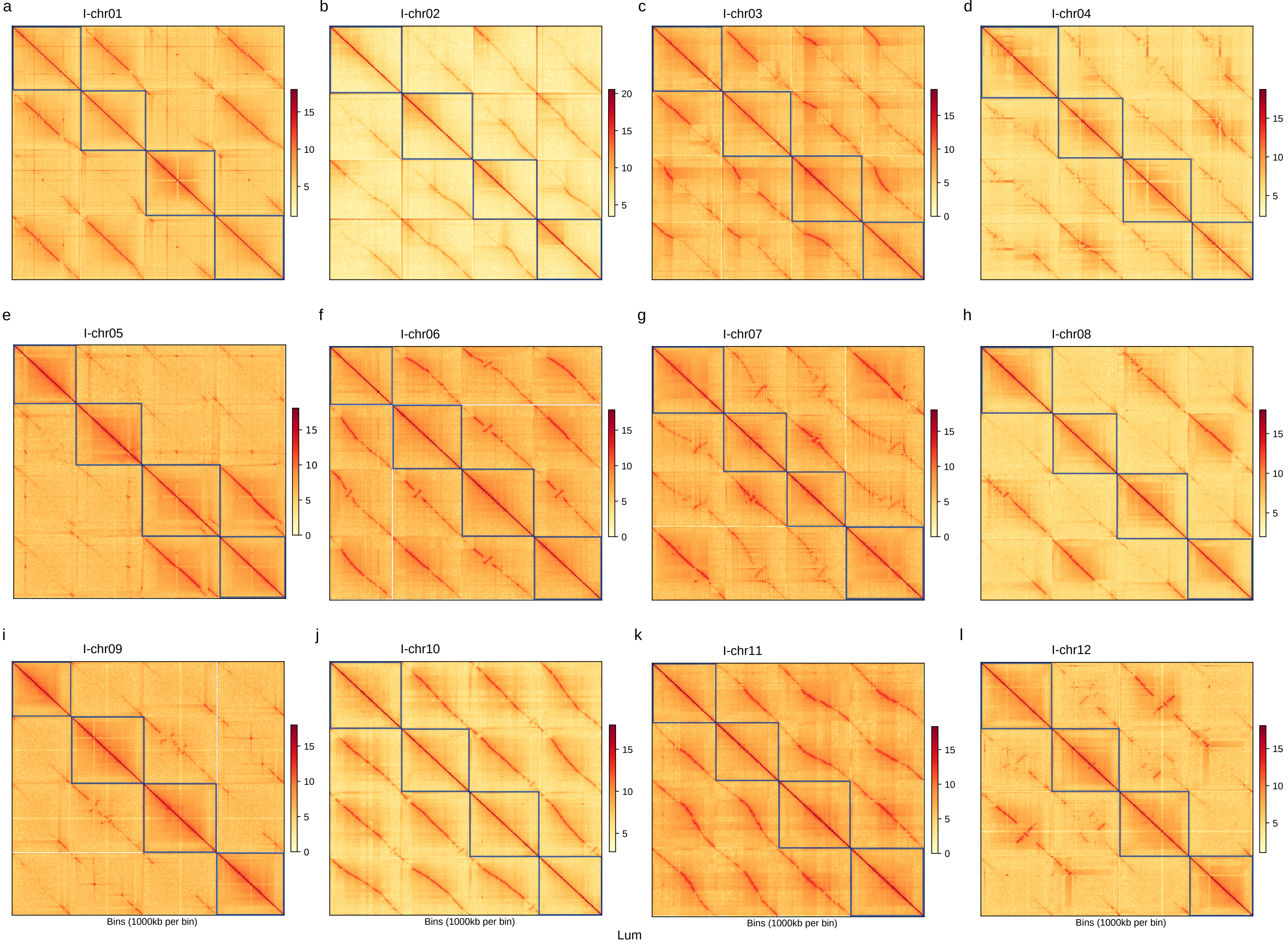
a
I-chr01
b
I-chr02
c
I-chr03
d
I-chr04
e
I-chr05
f
I-chr06
g
I-chr07
h
I-chr08
i
I-chr09
j
I-chr10
k
I-chr11
l
I-chr12
Bins (1000kb per bin)
Bins (1000kb per bin)
Bins (1000kb per bin)
Bins (1000kb per bin)
Lum
